# Lower Cerebrospinal Fluid/Plasma Fibroblast Growth Factor 21 (FGF21) Ratios and Placental FGF21 Production in Gestational Diabetes

**DOI:** 10.1371/journal.pone.0065254

**Published:** 2013-06-03

**Authors:** Bee K. Tan, Kavitha Sivakumar, Muhammad F. Bari, Manu Vatish, Harpal S. Randeva

**Affiliations:** 1 Divisions of Metabolic & Vascular Health and Reproduction, Warwick Medical School, University of Warwick, Coventry, United Kingdom; 2 Nuffield Department of Obstetrics & Gynaecology, University of Oxford, Oxford, United Kingdom; Virgen Macarena University Hospital, School of Medicine, Spain

## Abstract

**Objectives:**

Circulating Fibroblast Growth Factor 21 (FGF21) levels are increased in insulin resistant states such as obesity, type 2 diabetes mellitus and gestational diabetes mellitus (GDM). In addition, GDM is associated with serious maternal and fetal complications. We sought to study human cerebrospinal fluid (CSF) and corresponding circulating FGF21 levels in women with gestational diabetes mellitus (GDM) and in age and BMI matched control subjects. We also assessed FGF21 secretion from GDM and control human placental explants.

**Design:**

CSF and corresponding plasma FGF21 levels of 24 women were measured by ELISA [12 GDM (age: 26–47 years, BMI: 24.3–36.3 kg/m^2^) and 12 controls (age: 22–40 years, BMI: 30.1–37.0 kg/m^2^)]. FGF21 levels in conditioned media were secretion from GDM and control human placental explants were also measured by ELISA.

**Results:**

Glucose, HOMA-IR and circulating NEFA levels were significantly higher in women with GDM compared to control subjects. Plasma FGF21 levels were significantly higher in women with GDM compared to control subjects [234.3 (150.2–352.7) *vs.* 115.5 (60.5–188.7) pg/ml; *P*<0.05]. However, there was no significant difference in CSF FGF21 levels in women with GDM compared to control subjects. Interestingly, CSF/Plasma FGF21 ratio was significantly lower in women with GDM compared to control subjects [0.4 (0.3–0.6) *vs.* 0.8 (0.5–1.6); *P*<0.05]. FGF21 secretion into conditioned media was significantly lower in human placental explants from women with GDM compared to control subjects (*P*<0.05).

**Conclusions:**

The central actions of FGF21 in GDM subjects maybe pivotal in the pathogenesis of insulin resistance in GDM subjects. The significance of FGF21 produced by the placenta remains uncharted and maybe crucial in our understanding of the patho-physiology of GDM and its associated maternal and fetal complications. Future research should seek to elucidate these points.

## Introduction

Pregnancy is a state of insulin resistance associated with profound changes in metabolism, aimed at supplying adequate nutrition to the fetus [1]. Gestational diabetes mellitus (GDM) and maternal obesity affects up to 20% of all pregnancies [2]–[5] and is associated with adverse maternal and fetal outcomes [6]–[8]. In particular, GDM is associated with an increased risk of developing obesity, type 2 diabetes mellitus (T2DM) and cardiovascular disease in both mother and child later on in life [7].

FGF21 has been recently described as a metabolic regulator predominantly produced in the liver as well as adipose tissue and has been shown to be an important factor in the homeostatic mechanisms with positive effects on glucose and lipid metabolism [9]. FGF21 levels were positively associated with obesity and the metabolic syndrome and increased in T2DM [10]–[14]. Furthermore, FGF21 has been reported to alleviate obesity in mice [15].

The hypothalamus is the key regulatory center for energy balance and is abundant with peptides that regulate satiety [16]. Many of these peptides are also produced in peripheral sites, in the case of FGF21, as mentioned above, the liver and adipose tissue. Recently, an elegant study by Hsuchou et al. had demonstrated that FGF21 crosses the blood-brain barrier (BBB) in mice and affirmed that peripheral FGF21 could reach the brain directly and thus potentially exert its central effects [17], [18]. Importantly, a recent report by Sarruf et al. had demonstrated that intracerebroventricular infusion of FGF21 in rodents promotes insulin sensitivity secondary to increased insulin-induced suppression of hepatic glucose production [18]. More recently, we have reported the existence of FGF21 in human CSF [19].

Given the above, we hypothesized that central FGF21 levels may be altered in women with GDM, which could impact upon, in particular, the dysfunctional glucose metabolism observed in women with GDM [20]; it has been reported that insulin-induced suppression of hepatic gluconeogenesis in women with GDM is impaired [20]. Also, it is plausible that FGF21 could influence fetal metabolism and account for the metabolic complications found in babies born to women with GDM. In relation this, it had been reported that adipokines such as leptin, resistin and adiponectin are produced by the placenta and that changes in the secretion of these adipokines could affect fetal growth and development [21]. The aim of this study was to investigate human cerebrospinal fluid (CSF) and corresponding circulating FGF21 levels in women with GDM and in age and BMI matched control subjects. We also assessed FGF21 secretion from GDM and control human placental explants.

## Materials and Methods

### Ethics

The study was approved by the Local Research Ethics Committee (Research Ethics Committees 07/H1210/141) and written informed consent was obtained from all participants, in accordance with the guidelines in The Declaration of Helsinki 2000.

### Subjects

We investigated 24 Caucasian women i.e. 12 diet controlled GDM {age: 33.5 [median] (30.5–39.0) [interquartile range] years; BMI: 32.7 [median] (27.6–35.2) [interquartile range] kg/m^2^)} and 12 controls {age: 34.0 [median] (28.5–34.5) [interquartile range] years; BMI: 31.0 [median] (30.2–32.2) [interquartile range] kg/m^2^)}. All study participants were pregnant women scheduled for elective caesarean section delivering at 39–40 weeks of gestation. GDM subjects were diagnosed by the 2-hour 75 g oral glucose tolerance test using the criteria defined by the World Health Organization i.e. fasting plasma venous glucose concentration greater than or equal to 7.0 mmol/l or 2-hour plasma venous glucose concentration greater than or equal to 7.8 mmol/l. Exclusion criteria included a history of diabetes, cardiovascular disease such as congestive heart failure, liver or kidney disease, malignancy, signs of inflammation, multiple pregnancies, pre-eclampsia and any drugs influencing body weight like corticosteroids or contraceptives. Subjects undertook simultaneous sampling of blood and cerebrospinal fluid (1 ml) *via* lumbar puncture after local anesthesia (2 ml mepivacaine-HCL 1%) at elective caesarean section; placenta was also obtained. Blood samples were immediately centrifuged at 3000 g (Beckman Coulter DS-9623C). Plasma and CSF samples were prepared within 1 hour and stored at −80°C until assayed. All patients underwent anthropometric measurements.

### Biochemical and Hormonal Analysis

Assays for glucose and lipid profile were performed using a Roche modular automated chemical analyser (Roche Diagnostics Scandinavian, Bromma, Sweden). Insulin levels were measured using a commercially available ELISA kit (Invitrogen, Paisley, UK), according to manufacturer’s protocol. The estimate of insulin resistance by Homeostasis Model Assessment (HOMA-IR) score was calculated as previously described [22]. Non-esterified free fatty acids (NEFA) levels in plasma were determined using a commercially available kit (BioVision, Cambridge, UK), according to manufacturer’s protocol. Leptin levels were measured using a commercially available ELISA kit (R & D Systems, Abingdon, UK), according to manufacturer’s protocol; with an intra-assay coefficient of variation of 3.6%. FGF21 levels were measured using a commercially available ELISA kit (BioVendor, Oxford, UK) according to manufacturer’s protocol; with an intra-assay coefficient of variation of 3.6%.

### Placental Explant Culture

Placental tissue explants were cultured for 48 hours as previously described [23]. Briefly, chorionic villi were dissected out and carefully rinsed in sterile Dulbecco’s phosphate-buffered saline with calcium chloride and magnesium chloride (37°C; Sigma-Aldrich, Gillingham, UK) to remove maternal blood. The placental tissue was cut into pieces weighing ∼5 mg. Three such pieces were cultured in individual Costar Netwell (15 mm diameter, 74 µm mesh; Corning Incorporated, Corning, USA) supports in 1.5 ml of culture medium (CMRL-1066, 5% heat-inactivated fetal bovine serum, 100 IU/ml penicillin, 100 µg/ml streptomycin; Sigma-Aldrich, Gillingham, UK). The tissue was supported on the mesh in the liquid-gas interface. Cultures were maintained at 37°C in a humidified gas mixture of 5% CO_2_ and 95% air, and conditioned media aliquots were collected after 24 hours and immediately centrifuged at 1000 rpm for 5 minutes and supernatants were stored in −80°C until assayed.

### Statistical Analysis

Data were analysed by Mann-Whitney *U* test. Data are medians (interquartile range). All statistical analyses were performed using SPSS version 18.0 (SPSS, Inc., Chicago, USA). *P*<0.05 was considered significant.

## Results

**Figure 1:**
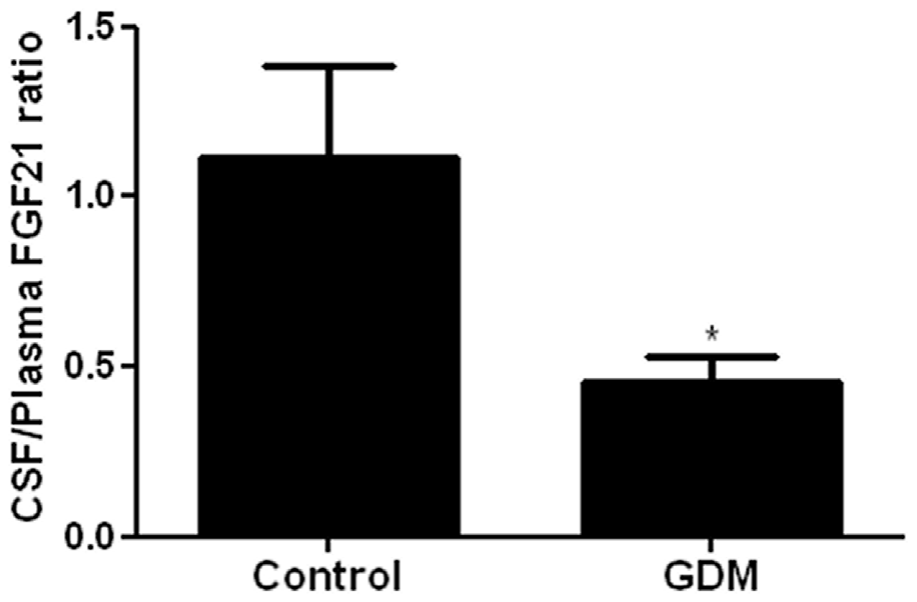
CSF/Plasma FGF21 ratio was significantly lower in women with GDM (*n = *14) compared to control subjects (*n = *12). Group comparison by Mann-Whitney *U* test. *P<0.05.

Table 1 shows the anthropometric, biochemical and hormonal data in all subjects. Glucose [4.5 (4.2–4.8) *vs.* 4.3 (4.0–4.7)nmol/µL; *P*<0.05], HOMA-IR [3.1 (1.8–5.3) pmol/L *vs.* 2.8 (1.0–5.1); *P*<0.05] and circulating NEFA levels were significantly higher in women with GDM compared to control subjects [2.2 (1.9–3.1) *vs.* 1.4 (1.1–1.5)nmol/µL; *P*<0.05]. Plasma FGF21 levels were significantly higher in women with GDM compared to control subjects [234.3 (150.2–352.7) *vs.* 115.5 (60.5–188.7)pg/ml; *P*<0.05]. However, there was no significant difference in CSF FGF21 levels in women with GDM compared to control subjects. Interestingly, CSF/Plasma FGF21 ratio was significantly lower in women with GDM compared to control subjects [0.4 (0.3–0.6) *vs.* 0.8 (0.5–1.6); *P*<0.05; Figure 1]. There was a non-significant positive association between plasma FGF21 and birth-weight (*P*<0.05).

**Table 1:** Clinical, hormonal and metabolic features of women with GDM and controls.

Variable	GDM (*n* = 12)	Controls (*n* = 12)	Significance
Age (year)	33.5 (30.5–39.0)	34.0 (28.5–34.5)	NS
BMI (kg/m^2^)	32.7 (27.6–35.2)	31.0 (30.2–32.2)	NS
Birth-weight (kg)	3.7 (3.6–3.8)	3.6 (3.4–3.8)	NS
Glucose (mmol/L)	4.5 (4.2–4.8)	4.3 (4.0–4.7)	*P*<0.05
Insulin (pmol/L)	107.0 (65.3–172.9)	100.4 (38.9–170.2)	NS
HOMA-IR	3.1 (1.8–5.3)	2.8 (1.0–5.1)	*P*<0.05
Total Cholesterol(mmol/L)	5.9 (5.7–6.4)	6.5 (5.5–7.5)	NS
HDL-Cholesterol(mmol/L)	1.4 (1.1–1.7)	1.7 (1.2–1.9)	NS
LDL-Cholesterol(mmol/L)	3.3 (2.9–4.3)	4.2 (3.1–4.7)	NS
Triglycerides(mmol/L)	2.8 (2.6–3.5)	2.7 (2.5–3.3)	NS
NEFA (nmol/µL)	2.2 (1.9–3.1)	1.4 (1.1–1.5)	*P*<0.05
Leptin (ng/ml)	43.0 (37.9–57.3)	42.1 (30.5–52.7)	NS
CSF FGF21(pg/ml)	96.2 (91.3–99.5)	93.1 (91.4–97.6)	NS
Plasma FGF21(pg/ml)	234.3 (150.2–352.7)	115.5 (60.5–188.7)	*P*<0.05
CSF/PlasmaFGF21 ratio	0.4 (0.3–0.6)	0.8 (0.5–1.6)	*P*<0.05

Data are medians (interquartile range). Group comparison by Mann-Whitney *U* test.

NS = not significant.

### FGF21 Secretion into Conditioned Media from GDM and Control Human Placental Explants

**Figure 2:**
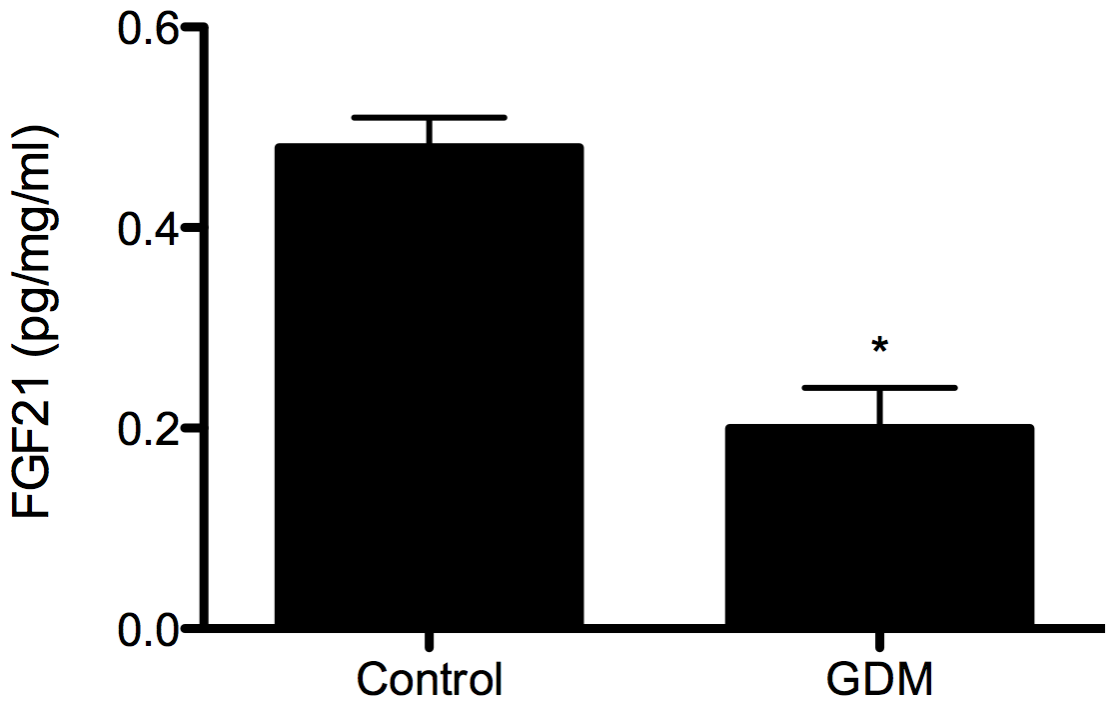
FGF21 levels in conditioned media of placental explants at 48 hours were assessed by ELISA. Four independent experiments were performed. Group comparison by Mann-Whitney *U* test. *P<0.05.

FGF21 secretion into conditioned media was significantly lower in human placental explants from women with GDM compared to control subjects (Figure 2). The clinical characteristics of the patients from whom the biopsies were taken are described in Table 2.

**Table 2:** Clinical characteristics of the women whose placentas were used for the explant studies. Subjects 1 to 4: Controls. Subjects 5 to 8: GDM.

Variable	Age (year)	BMI (kg/m^2^)	Parity	Gestational Age (week)
Subject 1	30	28	0	39+1
Subject 2	35	31	0	39+4
Subject 3	38	33	0	39+3
Subject 4	25	30	0	39
Subject 5	28	33	0	39
Subject 6	36	31	0	39+1
Subject 7	38	33	0	39+2
Subject 8	33	34	0	39+2

## Discussion

We present novel data of CSF and plasma FGF21 levels in women with GDM and matched (age, BMI) controls. Circulating FGF21 levels were significantly higher in women with GDM compared to controls. However, there were no significant differences in CSF FGF21 levels in women with GDM compared to controls. Importantly, the CSF/Plasma FGF21 ratio was significantly lower in women with GDM compared to control subjects.

Recently, a landmark study by Sarruf et al. had shown that continuous intracerebroventricular infusion of FGF21 in rats improves insulin sensitivity *via* increased insulin induced inhibition of hepatic gluconeogenesis [18]. It was proposed that FGF21 engages with fibroblast growth factor receptor-1, which is mainly expressed in the arcuate and ventromedial nuclei of the hypothalamus (areas of the hypothalamus that also mediate the central metabolic effects of insulin, leptin, oleate and glucose) and regulates gluconeogenesis [18].

In order for FGF21 to impose its central effects, circulating FGF21 should cross the blood-brain and/or blood-CSF barriers. In relation to this, Hsuchou et al. had surmised that FGF21 traverses the BBB by simple diffusion in mice [17]. Our finding of a significantly higher plasma FGF21 in the background of essentially similar CSF FGF21 levels leading to a lower CSF/Plasma FGF21 ratio in women with GDM compared to control subjects implies either that there is a deficiency of FGF21 transport across the BBB or that discrepancies in the production and/or metabolism of FGF21 by the central nervous system may explain the differences in CSF/plasma FGF21 ratio between the GDM and control subjects in our study. In relation to this, Yamashita et al. had reported that FGF23 is produced in the brain, particularly, by the ventrolateral thalamic nuclei [24]. It is thus plausible that FGF21 may also be produced in the brain. Furthermore, it is possible that the efficiency of FGF21 uptake into the CSF is decreased in GDM subjects possibly secondary to saturation of transporters. When saying this, we are mindful of the good evidence by Hsuchou et al. who had shown that FGF21 crosses the BBB non-saturably in mice [17]. However, there may be species differences in transport of FGF21 across the BBB. The coexistence of non-saturable and saturable mechanisms is also plausible given that other adipokines, e.g. leptin, have been shown to cross the BBB through both non-saturable and saturable mechanisms [25].

Taken together, the higher levels of plasma FGF21 in women with GDM could be a compensatory response to the underlying differences in maternal physiology such as higher insulin resistance at the start of the pregnancy [26] and/or poorer reserve in insulin secretion capacity in GDM subjects [27]. In relation to this, it has been reported that insulin-induced suppression of hepatic gluconeogenesis in women with GDM is impaired [20]. As mentioned above, given that intracerebroventricular infusion of FGF21 promotes insulin sensitivity by a similar mechanism [18], we hypothesize that the higher plasma FGF21 in the background of essentially similar CSF FGF21 levels (lower CSF/plasma FGF21 ratio) in GDM subjects may indicate a central failure in this compensatory response through either dysfunctional transport of circulating FGF21 through the BBB into the CSF or disordered metabolism of FGF21 within the CSF as discussed above. Therefore, the lower CSF/plasma FGF21 ratio in GDM subjects could be a marker of central FGF21 resistance in this context.

In addition, we report for the first time the secretion of FGF21 by the placenta. FGF21 produced in the placenta could enter the fetal circulation. FGF21 in the fetus akin to that in the adult may have favourable effects on fetal glucose and lipid metabolism [9]. We also found that the secretion of FGF21 from human placental explants of women with GDM was significantly lower compared to control subjects. There are two possibilities for this finding; firstly, that culturing GDM placentae in normal media significantly alters its secretion of FGF21 or secondly, that GDM placentae genuinely secrete less FGF21 than normal subjects. Lower placental FGF21 production in GDM subjects may translate to lower FGF21 action in the fetus and may account for the fetal metabolic complications observed in these women [7]. Further studies are needed to support this hypothesis.

A limitation of this study is that we have not assessed the presence of other members of the FGF family in human CSF. Moreover, we have not been able to measure the energy status in our study subjects. Given that intracerebroventricular infusion of FGF21 increased appetite and energy production in rats [18], it would be of interest to establish the relationship between the levels of other members of the FGF family in human CSF with FGF21 and to correlate this with the energy status of our study subjects. Furthermore, given ethical constraints i.e. CSF and blood samples could and were only obtained at the end stage of pregnancy, it is uncertain whether the CSF/plasma FGF21 ratio became divergent between GDM and control subjects before or after the development of GDM. Also, we have not been able to provide data on other adipokines (adiponectin, resistin, visfatin or vaspin) or placental lactogen/other placental hormones in these subjects. Finally, we have not been able to explore fully the role of FGF21 derived from the placenta on both mother and child.

In summary, the central actions of FGF21 in GDM subjects may be pivotal in the pathogenesis of insulin resistance in GDM subjects. The significance of FGF21 produced by the placenta remains by and large unexplored. Future research should seek to elucidate these points.
